# Changes in post-transplant serum testosterone levels in men undergoing lung transplantation: a pilot study using the TriNetX Research Network

**DOI:** 10.1038/s41443-024-00921-7

**Published:** 2024-06-05

**Authors:** Austin Thompson, Danly Omil-Lima, Jaime Abraham Perez, Erin Jesse, Mohit Khera, Kenneth Chavin, Nannan Thirumavalavan

**Affiliations:** 1 Case Western Reserve University School of Medicine, Cleveland, OH USA; 2 Fox Chase Cancer Center at Temple University, Philadelphia, PA USA; 3 Clinical Research Center, University Hospitals Cleveland Health System, Cleveland, OH USA; 4 University Hospitals Cleveland Medical Center, Department of Urology, Cleveland, OH USA; 5 Baylor College of Medicine, Scott Department of Urology, Houston, TX USA; 6 Department of Surgery, Lewis Katz School of Medicine at Temple University, Philadelphia, PA USA

**Keywords:** Gonadal disorders, Urogenital diseases

## Abstract

Hypogonadism is understudied in men requiring solid organ transplants, particularly among lung transplant recipients. Improvement in serum testosterone levels has been reported in kidney and liver transplantation. Using the TriNetX Research Network, we performed a retrospective cohort study to evaluate the incidence of peri-transplant hypogonadism and the natural course of serum testosterone following successful lung transplantation. Men aged ≥ 18 with a lung transplant and total testosterone drawn within one year pre- and post-transplant were included. Men with receipt of testosterone therapy were excluded. A low testosterone (<300 ng/dL) and normal testosterone (≥300 ng/dL) cohort was created before employing descriptive and analytic statistics to investigate the incidence of peri-transplant hypogonadism and the change in serum testosterone levels following lung transplantation. In our entire cohort, lung transplantation was not associated with a significant increase in post-transplant serum testosterone (329.86 ± 162.56 ng/dL pre-transplant and 355.13 ± 216.11 ng/dL post-transplant, p = 0.483). The number of men with low testosterone decreased by 9.8% following lung transplantation but was not significant, p = 0.404. In this pilot study, no significant change in the number of hypogonadal men nor serum testosterone levels was observed among men undergoing lung transplantation.

## Introduction

In 2022, lung transplantation was the fourth most common solid organ transplant performed in the United States, totaling 2,743 transplants [1]. Despite significant improvements in surgical technique [2], pre- and post-surgical management [3], patient selection [4, 5], and immunosuppressive regimen [6], acute allograft rejection and post-transplant morbidity and mortality rates remain high [7–9]. Although lung transplantation is a life-saving treatment option known to improve the quantity and quality of life for patients with end-stage lung disease (ELSD), lung transplantation had the lowest national patient survival rates and highest graft failure rates amongst commonly transplanted organs (kidney, liver, heart, lung) [1].

Once selected, successful transplantation also requires management of frailty and aging-associated dysfunction, including physical weakness, depression, sarcopenia, and systemic inflammation—all which have been shown to contribute to post-transplant outcomes [10].

For men requiring hepatic and renal transplantation, low testosterone levels have been associated with end-stage liver [11] and kidney disease [12]. In both, as disease severity worsens, testosterone levels decline [11, 12]. This is troubling as low testosterone is associated with worse outcomes, including poor allograft survival in renal transplants [13]. Low testosterone is also associated with impaired graft function and low-grade rejection following cardiac transplants [14]. Importantly, improvement in serum testosterone levels following solid-organ transplantation and resolution of chronic organ failure has been shown to reduce associated risk factors and improve sexual dysfunction following both renal [15] and hepatic transplants [16, 17].

The sequelae of hypotestosteronemia in males, such as decreased physical performance, osteoporosis, lethargy, and depression [18], may complicate transplant success. Testosterone also plays a vital role in the maintenance of normal respiratory function, and low serum levels have been associated with chronic respiratory failure and chronic obstructive pulmonary disease (COPD) [19]. However, in the setting of lung transplantation, the effect of low serum testosterone on outcomes around the time of transplant is poorly understood. To our knowledge, no published data exists on the incidence of low testosterone prior to and after lung transplantation. Thus, this pilot study seeks to evaluate the incidence of low serum testosterone in men prior to and after lung transplantation with the hypothesis that transplantation may improve testosterone levels.

## Materials and methods

### Study design

We conducted a retrospective cohort analysis utilizing electronic medical record (EMR) information retrieved from TriNetX Research Network (Cambridge, MA, USA). We used the International Classification of Disease (ICD-9 & 10, ICD-10-PCS), Current Procedural Terminology (CPT), and RxNorm codes to identify men for inclusion in this study. Supplemental Table 1 contains the complete list of ICD, CPT, and RxNorm codes used in this study. The STROBE (Strengthening the Reporting of Observational Studies in Epidemiology) guidelines for a cohort study were followed when writing this manuscript [20].

### Data source, patient selection, and outcomes

We compiled all clinical data from 11/16/2007 – 08/03/2021 using the TriNetX Research Network, a globally federated health research network (with a waiver from Western IRB) that provides de-identified clinical information from 58 health care organizations (HCOs), and over 80 million patients located within the United States. All data is sourced directly from the electronic medical record (EMR) of HCOs. Information relating to participating HCOs is also de-identified; however, a typical HCO includes a large academic healthcare system with inpatient, outpatient, and specialty care services.

We selected all adult male patients (aged ≥18) with a lung transplant, and total testosterone findings drawn within one year before or after lung transplant. If a patient had multiple testosterone measurements, we used the most recent pre-transplant testosterone lab and the latest post-transplant testosterone lab. We excluded all patients previously on testosterone therapy. We categorized patients into two groups, based on total testosterone level prior to transplant. We defined patients with total testosterone levels <300 ng/dL as having low testosterone (Low T), and patients with total testosterone levels ≥300 ng/dL as having normal testosterone (Normal T). For all patients, we collected clinical information including demographics, comorbidities, laboratory findings, and medication use (Table 1). Patients were followed until their last record in the TriNetX Research Network. Missing or partial data is not imputed or estimated by the TrinetX platform. Exceptions that may influence this study include discordance with HCO-supplied encounter dates and HCO encounter list. If an encounter is missing a start date, the earliest start data associated with the observation from the same encounter is used. If an encounter is missing an end date, the latest end date from the observations associated with the encounter is used. (https://trinetx.com/) Therefore, all patients included in this analysis should not have any missing data for our outcomes.

**Table 1:** Baseline characteristics and outcomes between patients with low total testosterone (Low T) and normal total testosterone (Normal T) levels before transplant.

Characteristic	Overall (N = 51)	Normal T (N = 25)	Low T (N = 26)	p-value
Age	55.5 ± 13.1	57.2 ± 13.8	53.8 ± 12.5	0.374
Race				
White	33 (64.7%)	14 (56.0%)	19 (73.1%)	0.202
Black or African American	3 (5.9%)	1 (4.0%)	2 (7.7%)	>0.999
Asian	2 (3.9%)	2 (8.0%)	0 (0.0%)	0.235
American Indian or Alaska Native	0 (0.0%)	0 (0.0%)	0 (0.0%)	
Native Hawaiian or Other Pacific Islander	0 (0.0%)	0 (0.0%)	0 (0.0%)	
Hispanic or Latino	2 (3.9%)	0 (0.0%)	2 (7.7%)	0.49
Comorbid conditions				
Multiple transplants	11 (21.6%)	6 (24.0%)	5 (19.2%)	0.679
History of dialysis	0 (0.0%)	0 (0.0%)	0 (0.0%)	
Diabetes	22 (43.1%)	10 (40.0%)	12 (46.2%)	0.657
Obesity	14 (27.5%)	3 (12.0%)	11 (42.3%)	0.015
COPD	20 (39.2%)	12 (48.0%)	8 (30.8%)	0.208
Restrictive lung disease	19 (37.3%)	10 (40.0%)	9 (34.6%)	0.691
Cystic fibrosis	4 (7.8%)	2 (8.0%)	2 (7.7%)	>0.999
Pulmonary vascular disease	4 (7.8%)	2 (8.0%)	2 (7.7%)	>0.999
Pulmonary hypertension	27 (52.9%)	13 (52.0%)	14 (53.8%)	0.895
Smoking history	27 (52.9%)	14 (56.0%)	13 (50.0%)	0.668
Medication use				
Immunosuppressant use	48 (94.1%)	25 (100.0%)	23 (88.5%)	0.235
Mycophenolate	1 (2.0%)	0 (0.0%)	1 (3.8%)	>0.999
Tacrolimus	14 (27.5%)	8 (32.0%)	6 (23.1%)	0.475
Methylprednisolone	47 (92.2%)	24 (96.0%)	23 (88.5%)	0.61
Basiliximab	36 (70.6%)	20 (80.0%)	16 (61.5%)	0.148
Outcomes				
Rejection	41 (80.4%)	21 (84.0%)	20 (76.9%)	0.726
Pneumonia	25 (49.0%)	12 (48.0%)	13 (50.0%)	0.886
Pulmonary embolism	2 (3.9%)	0 (0.0%)	2 (7.7%)	0.49
Pulmonary edema	8 (15.7%)	4 (16.0%)	4 (15.4%)	>0.999
Post-operative ECMO	11 (21.6%)	3 (12.0%)	8 (30.8%)	0.103
Mortality at 1 year	3 (5.9%)	2 (8.0%)	1 (3.8%)	0.61
Mortality at 3 years	8 (15.7%)	5 (20.0%)	3 (11.5%)	0.465
Mortality all time	11 (21.6%)	5 (20.0%)	6 (23.1%)	0.789

Chi-square or Fisher’s exact test utilized for categorical data. Independent t-test utilized for continuous data.

*COPD* chronic obstructive pulmonary disease, *ECMO* extracorporeal membrane oxygenation.

Our transplant-related outcomes of interest were 1-year mortality, 3-year mortality, all-time mortality, transplant rejection, pulmonary embolism, pulmonary edema, pneumonia, and post-operative extracorporeal membrane oxygenation (ECMO).

### Statistical analyses

**Fig. 1:**
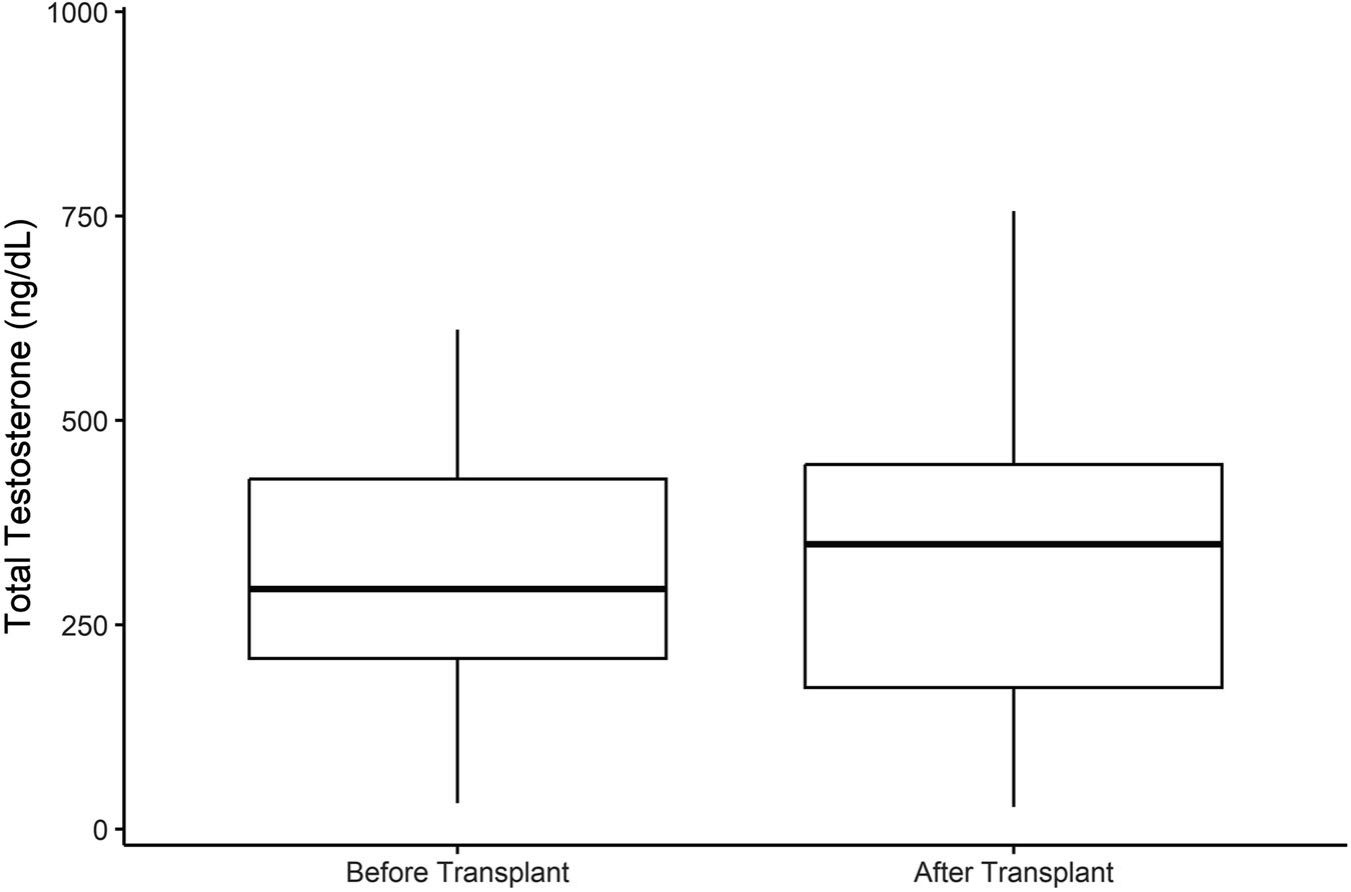
Box plot of median and interquartile range of total testosterone in the study population, before and after lung transplant.

We evaluated differences in baseline characteristics and outcomes between pre-transplant total testosterone levels (Normal T, or Low T), using chi-square or Fisher’s exact test for categorical data (presented as frequencies and percentages), and independent t-test for continuous data. We tested for changes in total testosterone before and after lung transplantation, using a paired t-test. Pre- and post-transplant total serum testosterone levels for the entire cohort were reported as the mean and standard deviation. Testosterone levels are represented as the median and interquartile range in Fig. 1.

We performed a sub-analysis where we further stratified patients by their pre-transplant total testosterone level (Low T, Normal T), and investigated changes in total testosterone within these two groups. A McNemar’s Test was used to assess the distribution of men with low and normal testosterone following lung transplantation. Analyses were performed using R version 4.2.1 (R Core Team, 2022) [21]. R code is available upon request.

## Results

### Patient characteristics

Using TriNetX, we retrieved a cohort of 51 men who underwent lung transplantation and had a testosterone level recorded one year prior to and after transplantation. Of these men, 25 had normal testosterone and 26 had low testosterone. Men with low testosterone had a significantly higher obesity rate compared to men with normal testosterone (42.3% vs. 12.0%, p = 0.015). Men with baseline low testosterone and normal testosterone did not statistically differ in additional demographic, medical comorbidities, medication use, and transplant-related outcomes as seen in Table 1.

### Association of lung transplantation and serum testosterone levels

The mean pre-transplant and post-transplant testosterone level for our entire cohort was not significantly different (329.86 ± 162.56 ng/dL vs. 355.13 ± 216.11 ng/dL, p = 0.483). The mean difference between the pre-transplant and post-transplant testosterone levels was 25.27 (-46.46, 96.99) ng/dL. Figure 1 represents the median and interquartile range for the pre- and post-transplant serum testosterone levels of the entire cohort.

### Association of lung transplantation and changes in the number of men with low testosterone

Prior to lung transplantation, 25 (49.0%) men had a normal testosterone level, and 26 (51.0%) had low testosterone. Following lung transplantation, 30 (58.8%) men had normal testosterone, and 21 (41.2%) had low testosterone χ^2^ = 0.696, p = 0.404.

## Discussion

Since the first lung transplant, multiple advances in the procedure have improved outcomes for patients requiring lung transplantation. Continuing to advance both the transplant procedure itself and the perioperative support is important, given a large volume of lung transplants are performed annually, with increases seen in the setting of COVID-19-related acute respiratory distress syndrome or fibrosis [22].

One possible avenue of improving transplant successes is focusing on sex-specific factors such as predominate hormones that maintain homeostatic integrity since biological differences can lead to disparity in solid-organ transplants [23]. With lung transplants, men are known to have worse long-term outcomes compared to women [24]. The influence of testosterone on transplant outcomes has been investigated in the renal [13] and heart [25] transplant literature. Although similar studies do not currently exist for lung transplantation, studies investigating the physiologic role of testosterone on lung function do exist. Hypotestosteronemia is common in men with COPD and correlates with worse lung function [26]. In women, increased levels of androgens are associated with worse lung function [27]. Whether this correlation of testosterone and lung function extrapolates to transplant patients remains unknown.

The role of testosterone recovery from the transplant itself or the use of exogenous testosterone supplementation has yet to be explored in the context of lung transplant outcomes. We know in men with hypogonadism, low testosterone is associated with erectile dysfunction, depression, obesity, decreased energy, decreased vigor, and physical performance [18, 28, 29]. Low testosterone also serves as a predictor for sarcopenia [30]. Interestingly, The International Society for Heart and Lung Transplantation (ISHLT), which establishes guidelines utilized by physicians in the selection process for lung transplant candidates, lists many of the known effects of hypotestosteronemia as contraindicates for transplant candidacy [31]. Although our study did not assess the impact of testosterone therapy in a pre-transplant setting, we did demonstrate that 51.0% of the men in our cohort were affected by low serum testosterone levels prior to surgery. In the post-transplant setting, testosterone therapy has been associated with prolonged survival in solid-organ recipients [32].

Our study demonstrated a non-statistically significant increase in mean serum testosterone levels in males undergoing lung transplantation. We observed a 9.8% reduction in the number of men with low testosterone following lung transplantation. However, the reduction in the number of men with low testosterone was not significant. Low testosterone levels persisted in 41.2% of men following transplant. Whether these findings translate to clinical improvement was not assessed in our study. However, improvement of post-transplant serum testosterone levels may offset the testosterone-lowering effects of transplant-related immunosuppression regimens. Immunosuppression medications are known to negatively impact serum testosterone levels and sperm counts [33]. Further studies are needed to assess the impact of testosterone recovery on lung transplant allograft rejection, infection rates, overall survival, and male reproductive capacity.

In the post-transplant setting, hypotestosteronemia has been associated with poor outcomes across multiple solid-organ transplants. For renal transplant recipients, hypotestosteronemia is associated with poor patient and allograft survival [13]. Impaired graft function and low-grade rejection are also reported following heart transplantation [14]. Similar complications, including allograft rejection [34, 35] and infection [34, 36] are seen in lung transplant patients. However, it is currently unknown if low serum testosterone levels influence the incidence or severity of post-transplant complications in lung transplant recipients. If the same association exists in lung transplantation, interventions to improve serum testosterone levels may reduce lung transplant complications while improving morbidity and mortality.

Many of the men in our study were affected by hypotestosteronemia prior to receiving a lung transplant. This finding aligns with data from other studies in the setting of renal and hepatic transplantation for patients with end-stage organ disease [11, 12]. In a previous review, we reported the occurrence of hypotestosteronemia in the setting of severe kidney, liver, and heart dysfunction [37]. Aberrations within the hypothalamic-pituitary-gonadal (HPG) axis are documented in patients with chronic kidney disease [38] and cirrhosis [39]. These studies represent organ-specific mechanisms leading to hypotestosteronemia. However, inflammation exists within heart failure [40], end-stage renal disease [41], chronic liver disease[42], and chronic obstructive pulmonary disease [43]. Increased pro-inflammatory cytokines seen with severe organ dysfunction may underlie the low serum testosterone levels seen across men requiring solid organ transplants [44].

Limitations inherent to the use of curated databases containing de-identified data impact our study. We used ICD-9/10, CPT, and RxNorm codes to identify patients, which may contain errors. The major limitation of our study is the small sample size. While TriNetX may be limited in the number of lung transplant patients included in the database, we created the largest cohort possible by retrieving patient health information from 58 healthcare organizations. Our data is also limited as TriNetX does not report the assay used or the collection time of testosterone labs. Finally, because medication doses are often reported as unknown, medications known to reduce testosterone levels, such as opioids, were not included in the analysis as the testosterone-lowering effect appears to vary with opioid dose and the medication duration of action [45]. Moreover, the risk of hypogonadism varies by medication [46]. Given the use of opioid medications for post-operative pain control, the post-operative testosterone levels observed in this study may be confounded by opioid use. However, opioid use for post-operative pain control is usually temporary and the rapid suppressive effect of morphine is reversible following discontinuation [47]. The results of this study are limited to men with known pre- and post-lung transplant serum testosterone levels in the United States.

Despite the aforementioned limitation, our work represents the first study to investigate testosterone levels in lung transplant patients. Prospective multi-institutional studies involving high-volume lung transplant centers may facilitate the creation of a large cohort and consistent morning testosterone collection that could better study the association between lung transplantation and serum testosterone levels. While analysis of perioperative opioid medications was not included in our study, prospective studies could also investigate the complex relationship that is opioid-induced androgen deficiency in the context of solid organ transplantation. In addition to investigating the impact of subsequent testosterone recovery following lung transplantation itself by excluding patients with testosterone therapy, the degree of pre-transplant hypotestosteronemia seen in our study also brings into question the impact of pre- and post-transplant testosterone therapy on lung transplant success. Future studies can address these questions.

Finally, future studies could implement age-stratified testosterone thresholds to further refine patient stratification and prognostication. However, universally agreed-upon age-stratified low testosterone thresholds do not currently exist, nor have they been implemented in the American Urologic Association guidelines. Research into the topic has begun. In young men, age-stratified low testosterone thresholds were higher than the current guideline-recommended threshold of 300 ng/dL [48]. Implementation of age-stratified low testosterone thresholds may therefore increase the number of peri-transplant hypogonadal men, which in the post-transplant setting, may prolong survival secondary to receipt of testosterone therapy treatment [32]. However, future research will be needed to expand age-stratified low testosterone thresholds to men aged 45 and above in addition to understanding if the transplant-related outcomes associated with the current definition of low testosterone are transferable to age-stratified thresholds.

## Conclusion

This pilot study showed no significant change in serum testosterone levels among men undergoing lung transplantation. 41.2% of our cohort remained hypogonadal after transplantation. Further research incorporating a larger cohort in a prospective study is required to determine which patients recover endocrine function after lung transplant and whether the etiology of chronic lung disease results in different outcomes after transplantation regarding serum androgen level and function.

## Supplementary information

Supplemental Table 1. Code and code descriptions for cohort criteria and study variables

## Data Availability

All data generated or analyzed during this study is included in this published article.
